# Factors Affecting Metabolic Outcomes Post Bariatric Surgery: Role of Adipose Tissue

**DOI:** 10.3390/jcm10040714

**Published:** 2021-02-11

**Authors:** Sara H. Keshavjee, Katherine J. P. Schwenger, Jitender Yadav, Timothy D. Jackson, Allan Okrainec, Johane P. Allard

**Affiliations:** 1 Vagelos College of Physicians & Surgeons, Columbia University, New York, NY 10032, USA; shk2176@cumc.columbia.edu; 2 Division of Gastroenterology, Toronto General Hospital, University Health Network, Toronto, ON M5G 2N2, Canada; kschweng@uhnresearch.ca; 3 Department of Immunology, University of Toronto, Toronto, ON M5S 1A8, Canada; jitu.yadav@mail.utoronto.ca; 4 Division of General Surgery, University Health Network, University of Toronto, Toronto, ON M5T 2S8, Canada; timothy.jackson@uhn.ca (T.D.J.); allan.okrainec@uhn.ca (A.O.)

**Keywords:** bariatric surgery, adipose tissue, metabolic outcomes

## Abstract

Obesity is an ever-growing public health crisis, and bariatric surgery (BS) has become a valuable tool in ameliorating obesity, along with comorbid conditions such as diabetes, dyslipidemia and hypertension. BS techniques have come a long way, leading to impressive improvements in the health of the majority of patients. Unfortunately, not every patient responds optimally to BS and there is no method that is sufficient to pre-operatively predict who will receive maximum benefit from this surgical intervention. This review focuses on the adipose tissue characteristics and related parameters that may affect outcomes, as well as the potential influences of insulin resistance, BMI, age, psychologic and genetic factors. Understanding the role of these factors may help predict who will benefit the most from BS.

## 1. Introduction

Obesity is an ever-growing problem and the World Health Organization estimated 650 million people to be obese in 2016 [1]. The main cause of obesity is the overconsumption of calories versus expenditure; however, other factors such as endocrine dysfunction, genetic makeup, and sleep debt can also contribute to obesity [2]. Along with obesity comes the burden of comorbidities, such as cardiovascular disease [3], diabetes [3], non-alcoholic fatty liver disease (NAFLD) [4], and hypertension (HTN) [5]. Specifically, obesity doubles the risk of HTN and triples the risk of type 2 diabetes mellitus (T2DM) in the 45–54 year old age category [6]. There is also a high prevalence of metabolic syndrome that accompanies obesity. Although criteria are controversial, someone is considered to have metabolic syndrome if they have three or more of the following: abdominal obesity, dyslipidemia, insulin resistance or hyperglycemia and HTN [7]. When compared to those of normal weight, individuals who are overweight have a 5.5-fold higher risk and individuals with obesity have a 32-fold higher risk of developing metabolic syndrome [8]. Despite this risk, not all individuals with obesity develop metabolic syndrome, and there is a stark contrast between those with “metabolically healthy” obesity verses “metabolically unhealthy” obesity. These relatively new categories of obesity came to light when noticing a subgroup of people with obesity, often early-onset, who have normal insulin sensitivity, and no signs of metabolic syndrome; “metabolically healthy” obesity [9]. Recently, the “metabolically healthy” obesity group has also been shown to have a decreased inflammatory state compared to similar-weight controls [10], and decreased liver fat content [11]. “Metabolically unhealthy” obesity is associated with a higher incidence of HTN, insulin resistance, and dyslipidemia, while “metabolically healthy” individuals with obesity have a lower risk of these comorbidities, along with a lesser degree of adipose tissue dysfunction [12].

One particularly effective treatment for obesity and its comorbidities is bariatric surgery (BS), which may be accomplished by various techniques such as Roux-en-Y gastric bypass (RYGB), sleeve gastrectomy (SG), and biliopancreatic diversion with duodenal switch (BPD) [13]. BS has been shown to be effective for achieving significant weight loss, with an average of 28.6% total body weight loss following RYGB and 25% following laparoscopic SG at five years post-surgery [14].

In addition to the direct benefits of weight loss, BS has been shown to decrease the magnitude of comorbidities such as HTN [15,16] and dyslipidemia, and even cause remission of T2DM [14,17,18]. The improvement on the three aforementioned comorbidities after RYGB surgery has been shown to be superior to medical and lifestyle management in a randomized controlled trial (RCT) of 120 patients [16]. Another RCT of 150 patients showed that an endpoint of HbA1c <6% without the use of diabetes medication was met by 29% of participants that received RYGB and by 23% who received SG, compared to only 5% for conventional medical therapy [19].

Despite huge successes in weight loss and comorbidity reduction from BS, not every patient achieves the significant weight loss and/or metabolic improvements. Approximately 10–20% of BS patients have insufficient weight loss one year after surgery, with excess body weight loss <40% or total weight loss <20% [20]. Additionally, even when patients initially achieve weight loss and improvements in T2DM, these problems can recur [21]. The challenge remains in predicting which patients will benefit most from BS. In this review, we will be summarizing the known predictors of BS outcomes, with a focus on the influence that adipose tissue characteristics may have.

## 2. Predictors of Bariatric Surgery Outcome

The success of bariatric surgery is generally evaluated based on percent total or percent excess body weight loss (EBWL) and reduction of comorbidities such as HTN, dyslipidemia, insulin resistance or T2DM. When attempting to predict the probability of BS success, studies often assess the severity of obesity (BMI, waist-to-hip ratio), age, and comorbidities at baseline. There are many other factors that are more recently being considered, such as social and psychological factors, as well as characteristics of the adipose tissue itself. This review focuses on adipose tissue characteristics and related parameters that may affect outcomes as well as the potential influence of insulin resistance, BMI, age, psychologic and genetic factors.

### 2.1. Adipose Tissue: Structure, Hypertrophy, Fibrosis

In obesity, as adipose tissue accommodates to caloric excess, it expands via hypertrophy or hyperplasia, to increase fatty acid storage [22]. This occurs in two maintain compartments: under the skin as the subcutaneous adipose tissue (SAT) or around abdominal organs as visceral adipose tissue (VAT). VAT includes the compartments of omental, retroperitoneal and mesenteric depots, each with varying metabolic properties [23]. Expansion of the visceral compartment is often associated with metabolically unhealthy obesity and development of comorbidities, whereas predominant SAT expansion is associated with metabolically healthy obesity [12]. Metabolic derangements and T2DM may be linked to VAT due to the fact that it is more metabolically active, and liver dysfunction can result from fatty acids, inflammatory cytokines and metabolites draining into portal circulation [24]. The visceral and subcutaneous fat is inherently different; subcutaneous fat confers fewer metabolic complications and may even be less harmful than VAT [25,26,27]. Adding to the complexity, even within one fat depot there are multiple subpopulations of adipocytes that have differing metabolic and physiological properties [28].

Clearly, expansion of adipose tissue is not benign in a patient with obesity. The depot of fat, along with method of expansion, has important effects on the development of comorbidities, including metabolic syndrome. Particularly, expansion of adipose tissue through hypertrophy can be indicative of dysfunctional adipocytes, inflammation, and risk for visceral adipose deposition [29]. Some studies have shown that the degree of adipocyte hypertrophy may predict increased risk of T2DM and a lower probability of T2DM remission after BS [30]. Hypertrophy, as opposed to hyperplasia, is also associated with worse metabolic derangements, such as dyslipidemia [31]. Thus, looking at adipocyte hypertrophy may provide important insight into the outcome of BS, especially in individuals with T2DM.

Additionally, during obesity, important changes occur in the extracellular matrix (ECM) of adipose tissue. As adipose tissue expands, the ECM is degraded; however, long-term inflammation, including macrophage infiltration [32], leads to a switch toward fibrosis, restricting adipose expansion [33]. This limitation in adipose expansion is thought to play a role in increasing visceral adipose deposition. Specifically, individuals with obesity show increased fibrosis [34] and expression of ECM component genes (such as various integrins, collagens, glycosaminoglycans and proteoglycans) in SAT compared to lean controls, along with a similar trend in liver fibrosis [35]. This may be mediated by a shift in adipocyte precursor population to a CD9+ phenotype, which has a pro-fibrotic effect [36]. There is, however, some evidence to show that fibrosis is not purely maladaptive, as VAT fibrosis may be protective against adipocyte hypertrophy and the consequent metabolic derangements, such as T2DM [37]. A study of 82 individuals undergoing BS found that SAT and VAT ECM deposition was decreased in both VAT and SAT of patients with T2DM, when observed by sirius red staining [37]. Consistent with the possible protective role of fibrosis, this study found that VAT fibrotic gene expression was decreased among diabetic patients, with a correlation to HbA1c levels [37]. Fibrosis may also be harmful, as another study of 65 patients found that those with increased SAT fibrosis had poorer fat mass loss percentage after RYGB surgery [31]. It is possible that despite fibrotic VAT’s protective effect on T2DM, there remains a detriment to weight loss following BS due to dysfunctional ECM remodeling. Overall, the fibrotic response may be indicative of the degree of inflammation and metabolic dysregulation, helping to predict outcomes after BS.

Changes in the adipose tissue architecture and lipid composition do occur after BS. One month following RYGB, patients’ VAT was found to have decreased fat fraction and increased T1 relaxation time on MRI in comparison to before surgery [38]. The T1 time is negatively correlated with fat content [39], showing that this may be a more nuanced measurement to take into account aside from adipose tissue mass, especially in regards to visceral adiposity. As soon as four weeks after BS, SAT adipocyte size has been shown to decrease significantly, along with a decrease in E2F1 expression, a marker of proliferation [40]. Interestingly, another study has shown that the number of adipocyte precursor cells is increased following BS and weight loss as compared to pre-BS, where numbers of precursors are often low in patients with obesity [41]. At two years post-RYGB, women’s fat cell volume was more closely associated with improved insulin sensitivity than reduction in fat mass [42]. This shows that the remodeling of the adipose tissue through fat content and adipocyte size may be a useful indicator of underlying metabolic changes, possibly returning the tissue to a metabolically healthy state. Associated changes may also occur in the ECM, as there is decreased expression of ECM component genes and increased expression of ECM degradation pathways (such as via metallopeptidases) in patients three months post-BS when compared to pre-operative levels [35]. Although gene expression shows anti-fibrotic changes after weight loss, there is debate as to whether existing fibrosis is reversible or not [35].

### 2.2. Adipose Tissue: Inflammatory Response

Numerous metabolic and inflammatory changes occur within expanding adipose tissue in a patient with obesity, which may contribute to development of comorbidities, including metabolic syndrome. As a result of the stress-induced changes, obesity has come to be considered a form of chronic inflammatory disease, driven by the close interactions of adipocytes and adipose tissue macrophages (ATM) [43]. These changes in adipose tissue are significant, and it is of interest to find out whether the varying inflammatory phenotypes of adipose tissue correlate to the varying outcomes of BS procedures.

Expansion of adipose tissue can lead to tissue hypoperfusion and hypoxia [44]. This makes hypoxic markers a promising area of research for predicting adipose tissue dysfunction. This hypoxia and adipose tissue stress causes a change in released adipokines, becoming more pro-inflammatory via IL-1β, IL-6, TNF, IL-8, leptin, resistin and MCP-1 [45]. This causes the recruitment of monocytes and as obesity progresses, ATM have been shown to progress from predominantly M2 to M1 type, assuming a more pro-inflammatory phenotype [46]. Aside from M1 and M2, some macrophages may take on an entirely different phenotype in obesity, the metabolically activated macrophage, which contributes to both inflammation and clearance of dead adipocytes [47].

The overall increase of inflammatory cytokines, as mentioned above, contributes to insulin resistance through a variety of mechanisms [48]. Specifically, expression of IL-6 from adipose tissue is elevated in obesity, with a threefold higher expression in omental fat as opposed to subcutaneous fat [49]. IL-6 can induce expression of C reactive protein (CRP), which are together used as clinical markers of inflammation and risk of T2DM development, independent of obesity [50,51]. TNF-α, produced by macrophages, has also been targeted as a link between obesity and insulin resistance, as a TNF-α antagonist Etanercept causes improvements in fasting blood glucose levels in obese individuals with metabolic syndrome [52]. TNF-α has also been shown to be an antagonist of GLUT4, a key mechanism of glucose uptake in response to insulin [53]. Additionally, MCP-1 expression has been shown to be elevated in SAT in obese individuals [54], along with elevations in those with T2DM without obesity [55]. MCP-1 likely has a direct role in insulin resistance, as mouse models with MCP-1 deletions are protected from high-fat diet induced insulin resistance and have less macrophage infiltration in adipose tissue [56]. However, MCP-1 may have broad-reaching affects, as MCP-1 knockout mice also had protection from hepatic steatosis during high-fat diet induced obesity [56]. Taken together, markers of inflammation along with insulin resistance measurements may help to predict chance of T2DM remission and outcomes after BS.

Many changes have been seen to occur in the inflammatory response following BS. At the tissue level, there is a decrease in subcutaneous ATMs after BS and weight loss, with an increase in IL-10 cytokine expression, signaling a shift to an anti-inflammatory M2 phenotype [57]. Multiple studies have shown a decrease in M1 and increase in M2 macrophages [58] appearing within three months post-BS [59]. However, the omental adipose tissue macrophages are likely the most important to assess, as they may be largely responsible for metabolic derangements. Cancello et al. found twice the number of ATMs in omental versus subcutaneous fat, with only omental macrophage counts correlating to insulin resistance and hepatic fibroinflammatory lesions [60]. Another contributing factor to the inflammatory profile improvement after BS is that hypoxic dysfunction of adipose tissue was shown to be reduced, including a reduction in hypoxic marker HIF-1α, macrophage chemo-attractants (MCP-1, CSF-3, PLAUR), and macrophage numbers [57]. On a systemic level, a meta-analysis of 116 studies showed that circulating levels of inflammatory markers like IL-6, TNF-α, and CRP significantly decreased following BS, which is in line with other studies on traditional weight loss [57]. Therefore, the reduction in inflammation following BS may be a product of the weight loss rather than the surgery itself [61,62].

The change in adipose tissue mass and degree of inflammation post-BS may be predicted by pre-surgical systemic and adipose-specific markers. When looking at predicting BS outcome, higher pre-operative systemic CRP levels have been shown to be associated with increased weight loss post-BS [63]. Another study found that high hs-CRP in women is able to predict the degree of reduction in visceral fat area one year post-surgery [64]. In general, a study of 37 patients undergoing BS found that an increase in adipose tissue inflammatory response (as measured by CD11b and IL-10 mRNA expression in adipose tissue) was associated with lower BMI loss after BS [65]. When looking broadly at the level of the serum proteome, Wewer Albrechtsen et al. found that 88 proteins changed significantly from baseline when measured again one week after surgery [66]. Many of the implicated proteins are important in inflammatory processes (complement, acute phase proteins, CRP) or lipid homeostasis [66]. Some proteins showed changes up to two years later, which may illustrate the difference between rapid surgery-induced effects and long-term weight loss-induced effects. Although the changes of inflammatory markers in response to BS have been researched, the role for many of these markers in predicting BS outcomes remains to be studied. Certain markers, such as circulating hs-CRP and tissue IL-10, may be implicated in predicting BS response, but there remains large potential for research regarding the use of inflammatory markers.

### 2.3. Adipose Tissue: Adipokine Dysregulation

As adipose tissue expands in obesity, the cells experience hypertrophy, along with oxidative and inflammatory stressors. This leads many individuals with obesity to experience altered adipokine secretion from adipose tissue, which can have far-reaching effects on the body and metabolism. In Table 1, we will briefly review the function of the main adipokines and their relationship to metabolic syndrome, followed by the effect of BS.

Along with the aforementioned adipokines in Table 1, many others have been studied and shown to have dysfunctional expression in obesity. These include visfatin [93], chemerin, lipocalin-2 [94], CXCL5, IL-18, and NAMPT [50], among others. Another area of interest regarding adipose tissue is that white adipose tissue, typically seen in obesity, [95] can change its phenotype into brown-like adipose tissue, called beige/brite adipose tissue, which is associated with improvements in IR, reduction in blood glucose and increased resting energy expenditure [96,97,98]. The increase in thermogenic capacity is mediated by UCP1 (uncoupling protein 1) [99,100], which is highly induced in brown-like adipocytes and expressed in the inner membrane of the mitochondria [95]. The transcription of UCP1 requires critical co-activators, specifically PCG1-α and PPARγ which commit the cells to thermogenesis [101,102]. This is of interest as recent research showed a decrease in functional brown adipose tissue in obesity [103].

In summary, analysis of adipokine levels pre-operatively may give insight into the degree of metabolic derangement and inflammation that is present in patients undergoing BS, while post-operative measures may track improvement in metabolic parameters. Although some adipokines have been shown to aid in BS outcome prediction as mentioned above, the role of others remains unclear.

### 2.4. Insulin Resistance and Type 2 Diabetes Mellitus

T2DM is a common phenomenon in individuals with obesity, as there is an association between obesity and insulin resistance in skeletal muscle, liver, and adipose tissue [104], along with pancreatic beta cell dysfunction [105]. T2DM remission often occurs after BS, especially from surgeries with a malabsorptive component; the highest remission rates occur after BPD (95%) and second-highest from RYGB (75%) [106]. However, remission after surgery is less likely in patients with poor glycemic control and increasing time since diabetes diagnosis, likely due to more extensive pancreatic beta cell damage and dysfunction [19,107].

A common clinical test for insulin resistance is the homeostatic model assessment for insulin resistance (HOMA-IR). HOMA-IR is generally increased in individuals with increased weight or obesity, and it is associated with T2DM and cardiometabolic complications [108]. In those with obesity, high HOMA-IR is also associated with steatosis and liver fibrosis [109]. Additionally, the probability of T2DM remission in the long and short term after RYGB surgery can be estimated with a DiaREM score, taking into account age, HbA1c and diabetes medication use [110,111]. Through predicting insulin resistance severity, these scores may help predict BS-related improvements in glycemic control.

Within one week after RYGB, before significant weight loss, patients experience improvements in glucose homeostasis [112]. This may be attributed to hepatic insulin sensitivity increase, as seen in one study measuring this via basal glucose and basal hepatic insulin sensitivity index [112]. Quick changes in insulin sensitivity following surgery may be due to calorie restriction after surgery, leading to reduced hepatic fat and subsequent insulin sensitivity increases [112,113]. Within one week, there are also increases in postprandial GLP-1 secretion, which enhances pancreatic beta cell function by stimulating insulin release [114]. One study using diet-induced obese rats found that the response to GLP-1 agonists has been shown to predict the efficacy of RYGB on glucose tolerance [115]. A recent study in T2DM individuals undergoing RYGB surgery found that those who experienced T2DM remission one year post-RYGB had significantly higher pre-RYGB GLP-1 concentrations [116]. However, research is limited and the degree in which GLP-1 predicts metabolic success post-RYGB is still contested [117]. In addition to GLP-1, HOMA_IR score has also been shown to decrease as soon as two weeks after surgery [118]. In the longer term, peripheral insulin resistance has been shown to improve by three months post-BS [119]. This may be mediated in part by continually decreasing intramyocellular fat [120], causing increased insulin sensitivity of skeletal muscle [121]. In another study, MRI analysis showed hepatic fat was reduced below the pathological range by six months and pancreatic fat 12 months post-BS, further explaining the long-term improvement in insulin resistance [122]. The beneficial effects of BS on the liver extend past insulin resistance, as a meta-analysis of 15 studies found that non-alcoholic steatohepatitis was resolved in 69.5% of cases and steatosis was improved in approximately 91.6% of cases [123]. There is evidence that surgeries with a malabsorptive component, such as RYGB, have better outcomes in terms of diabetes remission and improved HOMA-IR score than simply restrictive surgery in both short and long term [19,124,125,126,127]. In summary, there appears to be a role for using diabetes status and time since diagnosis to predict remission following BS, but its role in predicting weight loss and other metabolic parameters following BS remains to be studied.

### 2.5. BMI, Pre-Operative Weight Loss

Pre-operative BMI does seem to be an important predictor of BS results. Using a database of over seventy-thousand BS recipients, pre-operative weight has been shown to account for a large portion, approximately 18.5%, of the variation seen in weight loss post-BS [128]. Additionally, a meta-analysis has shown that many studies observed a lower EBWL percentage in those with a higher pre-operative BMI [129]. These results may be due to the fact that those with higher BMI are more likely to have the burden of comorbidities [130], including metabolic syndrome [8]. Along with BMI, surgery type is a major predictor of weight loss outcomes, explaining approximately 44.8% of the variability in a study of patients receiving RYGB, adjustable gastric band, or SG [128]. Evidently, BMI may be a major predictor in explaining BS outcome, and together with surgery type, these factors may explain a large portion of outcome variability.

The use of preoperative weight loss as a mandatory criterion before BS is a widely debated topic, with evidence for and against its utility. A study of the Swedish national registry for BS (*n* = 9570) found that there was a strong positive association between pre- and post-operative weight loss, especially in those in the highest BMI quartile [131]. Other studies saw similar data, such as a study of 884 patients undergoing RYGB that found those who achieved 10% EBWL preoperatively, were more likely to attain the goal of 70% EBWL post-BS [132]. A recent study of 355 RYGB or SG recipients asked patients to maintain a low calorie diet with the goal of 8% EBWL for four weeks before BS [133]. Those who achieved ≥8% EBWL had significantly greater EBWL at three, six and twelve months post-BS than those who did not achieve 8% EBWL pre-operatively [133]. Although many studies show a positive association between pre- and post-operative weight loss, it is difficult to tease out the impact of the pre-operative weight loss itself versus individual reactions to caloric restriction, along with the surgical candidate selection factors associated with mandatory preoperative EBWL.

### 2.6. Age

Along with the aforementioned factors, the age of the patient has been shown to have an effect on BS outcomes. In addition to having a negative impact on weight loss, higher age carries a higher risk of intraoperative and post-operative complications, which must be factored into the risk-benefit analysis of recommending BS. Some studies have reported significantly more complications in the older age group compared to those below 60 [134]. However, a recent study on more than three thousand patients undergoing RYGB or laparoscopic SG found no increase in intra-operative or post-operative complications for the 60+ age group [135]. Other studies have also found that EBWL is negatively affected by increasing age, including a study of more than thirteen-hundred patients aged 18–65 undergoing RYGB or SG [136].

### 2.7. Psychological Factors

Although many predictors of surgery outcome are not modifiable, some of the psychiatric conditions that correlate with poor outcome can be controlled and ameliorated before surgery. Patients with psychiatric disorders before SG surgery, such as personality disorders, adjustment disorders or depression, have been shown to have worse outcomes than those without mental illness [137]. Importantly, worse outcomes have been shown even in individuals with past mood disorders and no current episode, highlighting the possible role of additional treatment and social support before and after surgery in any patient with current or past mental health concerns [138].

### 2.8. Genetic Factors

Recently, studies have begun to look at how an individual’s genotype may be able to predict their response to BS. Many genome wide association studies have shown that there are hundreds of heritable genes that correlate with phenotypes such as waist-to-hip ratio and BMI [139]. Some alleles are even associated with a more metabolically healthy obese picture, with decreased comorbidities such as HTN, T2DM and heart disease [140]. When trying to analyze whether single nucleotide polymorphisms (SNPs) are associated with weight loss after RYGB, a genome wide association study by Rinella et al. found that genetic variants clustering around the genes of PKHD1, HTR1A, GUCY1A2, NMBR, KCNK2 and IGF1R may be implicated [141]. These genes have previously been related to biological processes such as appetite, lipid and glucose homeostasis and early onset obesity [141]. Similar results were seen in a study by Aasbrenn et al. [142]. Paradoxically, Aasbrenn et al. also noticed that individuals genetically predisposed to “slimness” experienced significantly poorer weight loss after surgery, possibly signaling social rather than biological causes of obesity in these patients [142]. In the future, genetic testing for SNPs may be used to predict a portion of one’s variable outcome after BS.

## 3. Conclusions

BS and the associated weight loss improve many metabolic and inflammatory parameters associated with changes in adipose tissue, adipokine expression, inflammatory profile and glucose/lipid homeostasis. The adipose tissue phenotype itself is very closely linked to the comorbidities that develop in individuals with obesity and their response to BS. Among pre-BS factors that may predict outcomes post-BS, those that have been found to be significant from the adipose tissue are VAT/SAT fibrosis, circulating CRP, Cd11b and IL10 adipose tissue mRNA levels, and possibly circulating leptin. In addition, other significant factors include diabetes status and time since diagnosis, pre-operative weight loss, age and psychological disorders. There may be other factors that can predict post-BS response, including a variety of adipokines and inflammatory markers (both circulating and expressed in adipose tissue), but further studies will be required in order to determine their significance. In the future, clinicians can use pre-operative data to better predict patient outcomes post-BS, as well as determine the optimal treatment plan for their patients.

## Figures and Tables

**Table 1:** Adipokines implicated in obesity and changes noted post-bariatric surgery (BS).

Adipokine	Role in Obesity Pathogenesis	Changes after BS
Leptin	↑ with degree of adiposity [67], signals hypothalamus to ↑ energy expenditure, ↓ hunger [68]. Pro-inflammatory effect on macrophages (increased TNF-α and IL-6), induces a Th1 cell inflammatory phenotype, releasing IL-2 and IFN-γ [50]. Leptin resistance may occur in obesity, for a variety of reasons [69,70,71]. Patients with increased circulating leptin pre-BS have increased weight loss post-BS [64]. However, other smaller studies have not found the same association [72].	↓ systemic leptin concentrations following various types of BS and weight loss [58,73].
Adiponectin	Functions in anti-apoptotic signaling, ↓inflammation, ↓insulin sensitivity [74]. ↓macrophage activation, ↑IL-10 production [50]. Levels are inversely correlated with adiposity [75] and degree of dysfunction in adipocytes [76]. Serum adiponectin <4.0 µg/mL greatly increases the likelihood of metabolic syndrome [77], can also be correlated with probability of developing T2DM [78]. Study of BS recipients (n = 1570) found that pre-BS adiponectin levels were not useful in predicting BS outcomes [79].	↑ circulating adiponectin within three months post-BS [80]. ↑ adiponectin adipose tissue expression with levels similar to normal weight controls within two years post-BS [81].
Resistin	↑IR, ↑hepatic glucose production, ↑inflammation (via monocyte secretion of IL6, TNF) [50]. Role in IR due to its stimulation of SOC3 expression, which inhibits insulin signaling in the adipocyte [82] Some studies show levels to be predictive of T2DM progression [83,84], where others have shown it to be associated with increased BMI rather than insulin resistance [85,86]. Inflammatory role-correlates with circulating CRP, IL-6 and TNF-α levels in patients with inflammatory diseases or T2DM [87]. Dyslipidemia—positive correlation with VLDL, and negative with HDL [87].	↓ in SAT expression 12 months post-BS [81]. However, results are conflicting [73].
Retinol Binding Protein 4 (RBP4)	Main protein to which retinol (vitamin A) binds to in serum, produced by liver and adipose tissue. ↑IR, which may be due to RBPP4’s negative correlation with GLUT4, which is necessary to import glucose into cells to be metabolized [88] ↑ Serum levels in obesity, associated with metabolic syndrome and T2DM [89]. Adipose tissue expression levels associated with ↑ BMI, waist circumference, circulating RBP4 and HOMA IR [90]. Dysregulated levels (high or low) are predictive of T2DM risk, independent of BMI [91].	↓ RBP4 in 12 months following BS, with a correlation to decreases in fasting glucose, serum triglycerides, and weight [92].
